# Supplementary material for: Genome-wide identification, characterization and expression analysis of the DUF668 gene family in tomato
Source: PeerJ. 2024 Jun 18;12:e17537. doi: 10.7717/peerj.17537 (PMC11192028; doi:10.7717/peerj.17537)
Supplement: Figure S1 — Numbers in the box are the number of cis-elements indicated by different intensity colors and numbers. The stacked graph on the right side represents the total number of promoter elements in each category. [file peerj-12-17537-s001.docx]

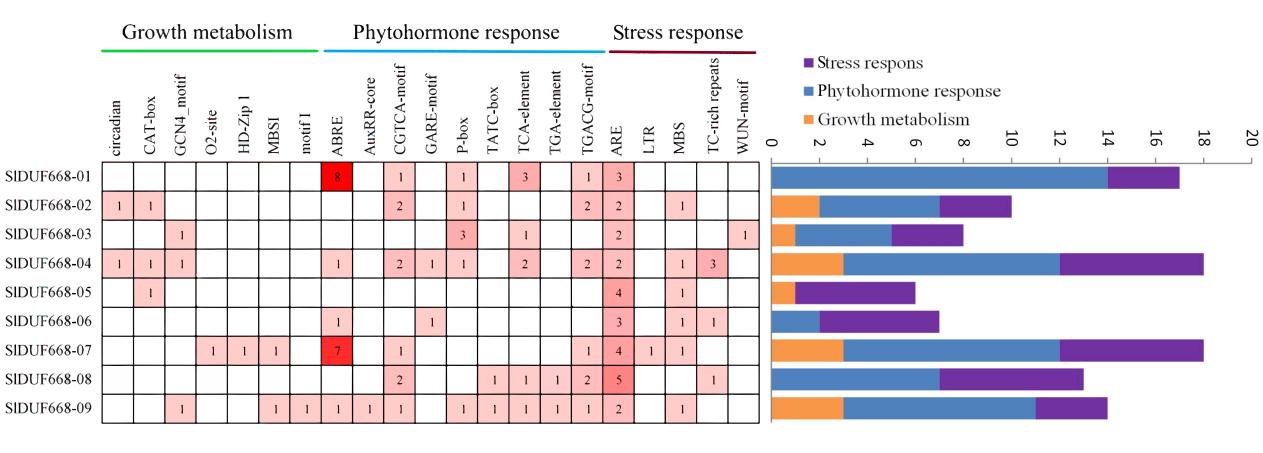


**Supplementary Fig. S1** Cis-acting elements analysis of the SlDUF668 gene family in tomato. Numbers in the box are the number of cis-elements indicated by different intensity colors and numbers. The stacked graph on the right side represents the total number of promoter elements in each category.
