# Supplementary material for: Genome-wide identification, characterization and expression analysis of the DUF668 gene family in tomato
Source: PeerJ. 2024 Jun 18;12:e17537. doi: 10.7717/peerj.17537 (PMC11192028; doi:10.7717/peerj.17537)
Supplement: Table S5 [file peerj-12-17537-s006.docx]

| Supplementary Table S5 Conserved motifs in the amino acid sequence of SlDUF668 proteins. | | |
| --- | --- | --- |
| Motif | Width Multi level | Consensus sequence |
| 1 | 50 | TLGAAGLALHYANIIIQIEKJVARPHSVPPBARDDLYQMLPPSIKSALRS |
| 2 | 29 | LQTLYFADKEKTEAAILELLVGLNYJVRF |
| 3 | 29 | WKAEMEKILZWLVPLAHNTIKWHSERGFE |
| 4 | 41 | QKKEVKHLKEKSLWSRTLDEVVEKLVRIVFYIYARIKAVFG |
| 5 | 40 | VQKLVSKDMDELLRJAAAEKREELKIFAKEVSRLGKKCKD |
| 6 | 50 | GFASGTATKGNKIGILAFEVANTMSKLANLWQSLSEENIKKLKEEILPSE |
| 7 | 41 | HKYLKKEAELKMKKMERLVQYTAELYHELEALDELEQDYRR |
| 8 | 41 | KQVRIAKVSEVSSLLGRAGTAGLGKAVDVLDTLGSSMTNLN |
| 9 | 11 | PQWHNLDRYFE |
| 10 | 49 | MGGLCSRRANAENTTGRGIPHGNGHFNYGAGTVYQSRGLPPQANNDPMP |
